# Prediction of co-expression genes and integrative analysis of gene microarray and proteomics profile of Keshan disease

**DOI:** 10.1038/s41598-017-18599-x

**Published:** 2018-01-10

**Authors:** Sen Wang, Rui Yan, Bin Wang, Peiru Du, Wuhong Tan, Mikko J. Lammi, Xiong Guo

**Affiliations:** 1 School of Public Health, Health Science Center of Xi’an Jiaotong University; Key Laboratory of Trace Elements and Endemic Diseases, National Health and Family Planning Commission, Xi’an, Shaanxi China; 2 Department of Cardiology, the Second Affiliated Hospital, Health Science Center of Xi’an Jiaotong University, Xi’an, Shaanxi China; 3 Ordance Industrial Hygiene Research Institute, Xi’an, Shaanxi China; 4 Department of Integrative Medical Biology, University of Umeå, Umeå, Sweden

## Abstract

Keshan disease (KD) is a kind of endemic cardiomyopathy which has a high mortality. However, molecular mechanism in the pathogenesis of KD remains poorly understood. Serum samples were collected from 112 KD patients and 112 normal controls. Gene microarray was used to screen differently expressed genes. Genevestigator was applied to forecast co-expression genes of significant gene. iTRAQ proteomics analysis was used to verify significant genes and their co-expression genes. GO, COG, IPA and STRING were applied to undertake function categorization, pathway and network analysis separately. We identified 32 differentially expressed genes; IDH2, FEM1A, SSPB1 and their respective 30 co-expression genes; 68 differential proteins in KD. Significant proteins were categorized into 23 biological processes, 16 molecular functions, 16 cellular components, 15 function classes, 13 KD pathways and 1 network. IDH2, FEM1A, SSBP1, CALR, NDUFS2, IDH3A, GAPDH, TCA Cycle II (Eukaryotic) pathway and NADP repair pathway may play important roles in the pathogenesis of KD.

## Introduction

Keshan disease (KD) is a kind of endemic cardiomyopathy which has a high mortality. It was reported firstly in Keshan County in northeast China in 1935, while the similar cases were found in Nagano Prefecture in Japan and northern mountains in North Korea in the 1950s^1^. It is characterized by acute or chronic episodes of heart disorder manifested by cardiogenic shock, arrhythmia, and congestive heart failure with cardiomegaly. The 2010 Statistical Yearbook of Health in China indicates that KD affected 40088 residents in 16 provinces^2^. However, molecular mechanism in the pathogenesis of KD remains poorly understood.

At first, there are many studies focused on the roles of selenium deficiency in the pathogenesis of KD. The levels of selenium in the soil and food of the endemic area are significantly lower than the ones in the non-endemic area. The levels of selenium in the blood and hair of the patients with KD are also significantly lower than those in the normal control individuals^3^.

Up to now, studies about the expression of abnormal genes in KD patients has become more and more popular. Previously, the different expression genes between the KD patients and normal controls were screened by our whole-genome microarray analysis^4^. Then the significant genes were screened out by SAM (significance analysis of microarrays) analysis. Fifty-nine genes were found to be up and nineteen genes down regulated. The function categories of the different genes contained metabolism, transcription, synthesis and modification of proteins, ion channels and transport proteins and cell-to-cell signal transduction^5^. In the previous study, nine different expression protein spots were observed between Keshan disease patients and health control ones in 2-dimensional gel electrophoresis images, of which eight were identified by MALDI-TOF-MS^6^.

The isobaric tags for relative and absolute quantitation (iTRAQ) labeling and coupled two-dimensional liquid chromatography tandem mass spectrometry (2D LC-MS/MS) have become important techniques in the quantitative proteomics technology due to the high quantitative accuracy and advantages of high throughput^7^.

In order to screen significant genes and proteins associated KD and build protein network in this study, the expression levels of the 78 differentially expressed genes observed in our previous KD whole genomics study^4^ were evaluated in the peripheral blood of KD patients and healthy controls via an oligonucleotide microarray analysis. Then, Genevestigator online tool was used to forecast the co-expression genes of the differently expressed genes. Finally, an iTRAQ-coupled 2D LC-MS/MS technique was employed to analyze the differently expressed protein in the sera of KD and normal individuals. Differently expressed proteins pathways and networks were analyzed using IPA (Ingenuity pathway analysis) and STRING (search tool for the retrieval of interacting genes) online systems separately. Besides KD, the approach of this study may also provide new insights to the underlying molecular mechanisms of other heart diseases.

## Results

### Microarray data analysis

The analysis of microarray data using the fold change criteria ≥3 and ≤0.33, revealed 32 up-regulated genes in KD compared to normal controls were noticed (Table 1). The fold changes ranged from 3.26 to 20.47. Notably, no down-regulated genes were observed.

**Table 1:** Differently expressed genes between KD patients and the normal controls.

Gene name	Description	Gene ID	Fold Change
TTC25	tetratricopeptide repeat domain 25	NM031421	20.47
RMND5A	required for meiotic nuclear division 5 homolog A	NM022780	14.23
IDH2	isocitrate dehydrogenase (NADP(+)) 2, mitochondrial	NM002168	13.24
A2ML1	alpha-2-macroglobulin like 1	NM_144670	10.91
FBXO15	F-box protein 15	NM152676	9.32
ZDHHC2	zinc finger DHHC-type containing 2	NM_016353	7.74
LEF1	lymphoid enhancer binding factor 1	NM016269	7.61
FEM1A	fem-1 homolog A	NM018708	7.12
CD3G	CD3g molecule	NM000073	6.57
SSBP1	single stranded DNA binding protein 1	NM003143	6.53
RASD1	ras related dexamethasone induced 1	NM-016084	6.28
SIGLEC8	sialic acid binding Ig like lectin 8	NM014442	5.21
ABCC13	ATP binding cassette subfamily C member 13	NR_003087	5.11
DIO3	iodothyronine deiodinase 3	NM001362	5.1
PDE8B	phosphodiesterase 8B	NM003719	4.82
TNFSF11	tumor necrosis factor superfamily member 11	NM-003701	4.77
TNNT2	troponin T2, cardiac type	NM000364	4.72
THBS1	thrombospondin 1	NM003246	4.7
ABI3BP	ABI family member 3 binding protein	NM-015429	4.69
MGAT3	mannosyl (beta-1,4-)-glycoprotein beta-1,4-N-acetylglucosaminyltransferase	NM002409	4.68
EYA4	EYA transcriptional coactivator and phosphatase 4	NM004100	4.67
SEZ6L2	seizure related 6 homolog like 2	NM201575	4.64
NUCKS1	nuclear casein kinase and cyclin dependent kinase substrate 1	NM022731	4.3
IGF2BP2	insulin like growth factor 2 mRNA binding protein 2	NM006548	3.93
KRR1	small subunit processome component homolog	NM_007043	3.85
GUCD1	guanylyl cyclase domain containing 1	NM_031444	3.82
ACSL6	acyl-CoA synthetase long-chain family member 6	NM001009185	3.61
C18orf10	tubulin polyglutamylase complex subunit 2	NM_015476	3.43
REXO2	RNA exonuclease 2	NM015523	3.40
MYBPC3	myosin binding protein C, cardiac	NM000256	3.39
EFNA1	ephrin A1	NM004428	3.28
CYP2B6	cytochrome P450 family 2 subfamily B member 6	NM000767	3.26

### qRT-PCR validation of microarray data

To confirm the differential expression of the genes revealed by microarray analysis, the genes of IDH2, FEM1A and SSBP1, which had different magnitudes of fold changes of gene expression, were selected for the assessment by qRT-PCR. The up-regulated expressions of these three genes were in accordance with those obtained from the microarray analysis (Figure S1).

### Clusters of co-expressed genes according to tissue or organ

**Figure 1:**
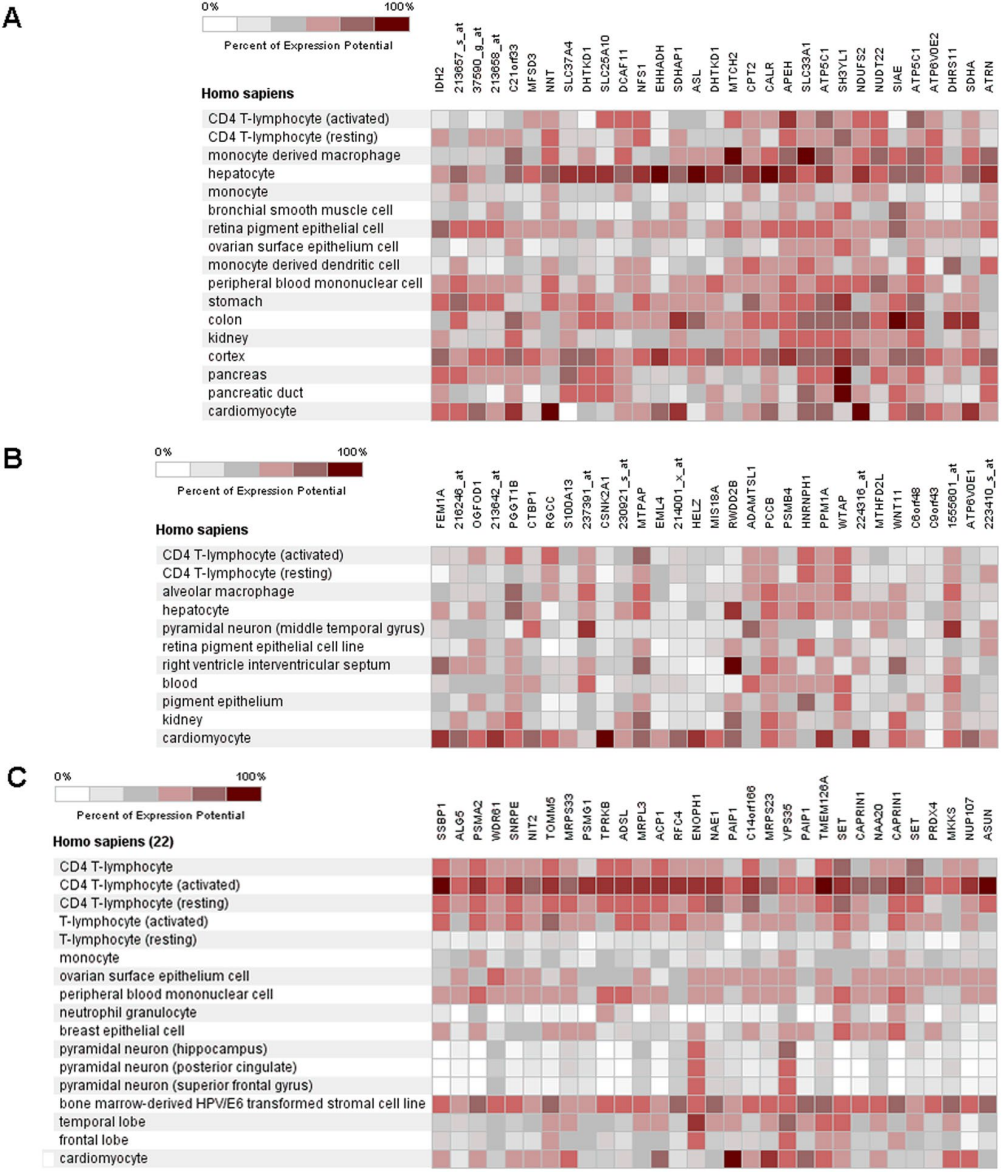
Three target genes and their predicted co-expressed genes screened by Genevestigator. (**A**) IDH2 (**B**). FEM1A and (**C**). SSPB1 and their 30 most co-expressed genes in different tissues and cells.

Hierarchical clustering of IDH2 (Fig. 1A), FEM1A (Fig. 1B) and SSPB1 (Fig. 1C) showing their 30 most co-expressed genes were screened by using Genevestigator tool. The color rectangles from white to red showed the percent of the gene expression potential. The genes expressed in the peripheral blood mononuclear cell or cardiomyocyte were considered as significant co-expressed genes.

### Protein identification and quantification

A total of 3134 unique peptides recognizing in 787 proteins were detected by iTRAQ proteomic analysis. We identified 68 significant differentially expressed proteins (Table 2), which had relative ratios higher than 1.5 or lower than 0.67. Among them, 49 proteins (72.1%) were up-regulated, while 19 proteins were down-regulated (27.9%).

**Table 2:** Differently expressed proteins between KD patients and the normal controls.

UniProtKB accession	Protein name	Gene name	Fold change
sp|P02042|HBD_HUMAN	Hemoglobin subunit delta	HBD	6.02
sp|P06401|PRGR_HUMAN	Progesterone receptor	PGR	4.02
sp|P12004|PCNA_HUMAN	Proliferating cell nuclear antigen	PCNA	3.85
sp|P35228|NOS2_HUMAN	Nitric oxide synthase, inducible	NOS2	3.41
sp|Q9Y251|HPSE_HUMAN	Heparanase	HPSE	3.18
sp|P24941|CDK2_HUMAN	Cyclin-dependent kinase 2	CDK2	3.06
sp|P02787|TRFE_HUMAN	Serotransferrin	TF	2.72
sp|Q04206|TF65_HUMAN	Transcription factor p65	RELA	2.68
sp|P10415|BCL2_HUMAN	Apoptosis regulator Bcl-2	BCL2	2.55
sp|P00738|HPT_HUMAN	Haptoglobin	HP	2.52
sp|P07288|KLK3_HUMAN	Prostate-specific antigen	KLK3	2.48
sp|P01100|FOS_HUMAN	Proto-oncogene c-Fos	FOS	2.44
sp|P02763|A1AG1_HUMAN	Alpha-1-acid glycoprotein 1	ORM1	2.43
sp|Q9Y3I1|FBX7_HUMAN	F-box only protein 7	FBXO7	2.38
sp|P04406|G3P_HUMAN	Glyceraldehyde-3-phosphate dehydrogenase	GAPDH	2.34
sp|P23528|COF1_HUMAN	Cofilin-1	CFL1	2.29
sp|P02776|PLF4_HUMAN	Platelet factor 4	PF4	2.26
sp|P25311|ZA2G_HUMAN	Zinc-alpha-2-glycoprotein	AZGP1	2.22
sp|P55072|TERA_HUMAN	Transitional endoplasmic reticulum ATPase	VCP	2.18
sp|P13798|ACPH_HUMAN	Acylamino-acid-releasing enzyme	APEH	2.17
sp|P10720|PF4V_HUMAN	Platelet factor 4 variant	PF4V1	2.09
sp|P03372|ESR1_HUMAN	Estrogen receptor	ESR1	2.05
sp|Q9BSK4|FEM1A_HUMAN	Protein fem-1 homolog A	FEM1A	1.97
sp|Q86UX7|URP2_HUMAN	Fermitin family homolog 3	FERMT3	1.95
sp|P08123|CO1A2_HUMAN	Collagen alpha-2(I) chain	COL1A2	1.94
sp|Q9Y490|TLN1_HUMAN	Talin-1	TLN1	1.93
sp|Q04837|SSBP_HUMAN	Single-stranded DNA-binding protein, mitochondrial	SSBP1	1.91
sp|P02788|TRFL_HUMAN	Lactotransferrin	LTF	1.85
sp|P02790|HEMO_HUMAN	Hemopexin	HPX	1.84
sp|P07996|TSP1_HUMAN	Thrombospondin-1	THBS1	1.84
sp|P01773|HV312_HUMAN	Immunoglobulin heavy variable 3–30	IGHV3–30	1.8
sp|P48735|IDHP_HUMAN	Isocitrate dehydrogenase [NADP], mitochondrial	IDH2	1.78
sp|P30041|PRDX6_HUMAN	Peroxiredoxin-6	PRDX6	1.76
sp|P27797|CALR_HUMAN	Calreticulin	CALR	1.75
sp|P50213|IDH3A_HUMAN	Isocitrate dehydrogenase [NAD] subunit alpha, mitochondrial	IDH3A	1.72
sp|P05164|PERM_HUMAN	Myeloperoxidase	MPO	1.7
sp|Q14766|LTBP1_HUMAN	Latent-transforming growth factor beta-binding protein 1	LTBP1	1.69
sp|Q13201|MMRN1_HUMAN	Multimerin-1	MMRN1	1.67
sp|P01011|AACT_HUMAN	Alpha-1-antichymotrypsin	SERPINA3	1.64
sp|A2BDB0|ACTG_XENLA	Actin, cytoplasmic 2	ACTG1	1.63
sp|P04264|K2C1_HUMAN	Keratin, type II cytoskeletal 1	KRT1	1.6
sp|O75306|NDUS2_HUMAN	NADH dehydrogenase [ubiquinone] iron-sulfur protein 2, mitochondrial	NDUFS2	1.6
sp|P01857|IGHG1_HUMAN	Ig gamma-1 chain C region	IGHG1	1.58
sp|P02774|VTDB_HUMAN	Vitamin D-binding protein	GC	1.57
sp|P01009|A1AT_HUMAN	Alpha-1-antitrypsin	SERPINA1	1.56
sp|P02549|SPTA1_HUMAN	Spectrin alpha chain, erythrocytic 1	SPTA1	1.56
sp|Q8WWA0|ITLN1_HUMAN	Intelectin-1	ITLN1	1.55
sp|P55083|MFAP4_HUMAN	Microfibril-associated glycoprotein 4	MFAP4	1.52
sp|Q8TDL5|BPIB1_HUMAN	BPI fold-containing family B member 1	BPIFB1	1.5
sp|P36980|FHR2_HUMAN	Complement factor H-related protein 2	CFHR2	0.66
sp|P55103|INHBC_HUMAN	Inhibin beta C chain	INHBC	0.65
sp|P20742|PZP_HUMAN	Pregnancy zone protein	PZP	0.65
sp|Q13103|SPP24_HUMAN	Secreted phosphoprotein 24	SPP2	0.64
sp|P04278|SHBG_HUMAN	Sex hormone-binding globulin	SHBG	0.63
sp|P26572|MGAT1_HUMAN	Alpha-1,3-mannosyl-glycoprotein 2-beta-N-acetylglucosaminyltransferase	MGAT1	0.61
sp|P00352|AL1A1_HUMAN	Retinal dehydrogenase 1	ALDH1A1	0.59
sp|Q96KN2|CNDP1_HUMAN	Beta-Ala-His dipeptidase	CNDP1	0.58
sp|Q92496|FHR4_HUMAN	Complement factor H-related protein 4	CFHR4	0.57
sp|P05543|THBG_HUMAN	Thyroxine-binding globulin	SERPINA7	0.57
sp|P29622|KAIN_HUMAN	Kallistatin	SERPINA4	0.56
sp|O14791|APOL1_HUMAN	Apolipoprotein L1	APOL1	0.52
sp|P08603|CFAH_HUMAN	Complement factor H	CFH	0.49
sp|Q9UGM5|FETUB_HUMAN	Fetuin-B	FETUB	0.47
sp|P01019|ANGT_HUMAN	Angiotensinogen	AGT	0.46
sp|P55056|APOC4_HUMAN	Apolipoprotein C-IV	APOC4	0.42
sp|P06312|KV401_HUMAN	Immunoglobulin kappa variable 4–1	IGKV4–1	0.42
sp|P08519|APOA_HUMAN	Apolipoprotein(a)	LPA	0.41
sp|Q02156|KPCE_HUMAN	Protein kinase C epsilon type	PRKCE	0.31

### Functional categorization, pathway and network analysis

**Figure 2:**
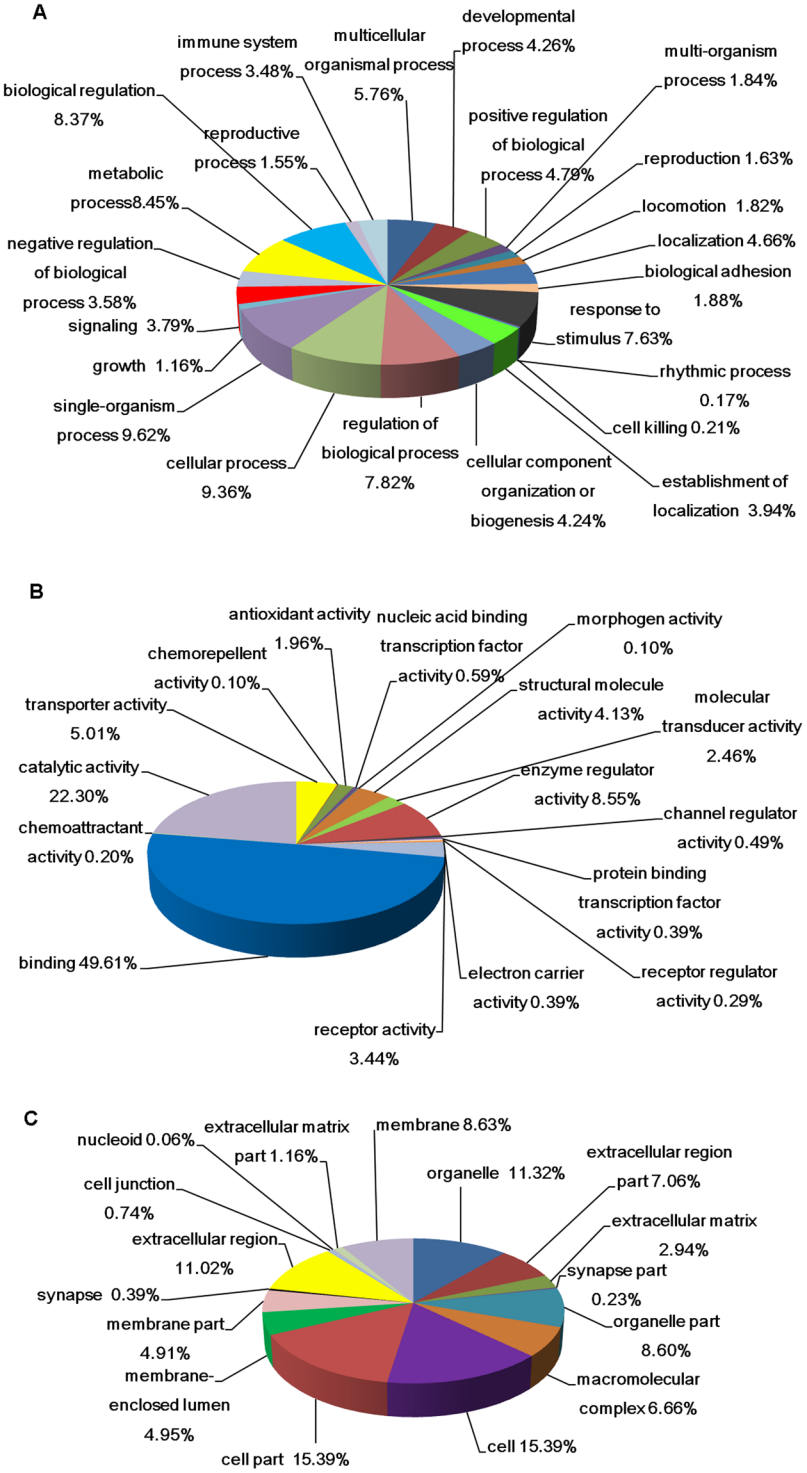
GO annotation of differential expression proteins in three domains: (**A**) biological process; (**B**) molecular function and (**C**) cellular component. The sectors of different colors show the percentage of each item.

Among the 68 differently expressed expression proteins, the proteins were categorized and displayed in the way of percentage by GO in three domains:23 biological processes (Fig. 2A), 16 molecular functions (Fig. 2B) and 16 cellular components (Fig. 2C). Fifteen function classes were categorized by COG. The function classifications and the number percentages of the protein in each classification were shown as follows: inorganic ion transport and metabolism (12%); cell motility (10%); translation, ribosomal structure and biogenesis (9%); nucleotide transport and metabolism (8%); cell wall/membrane/envelope biogenesis (8%); signal transduction mechanisms (8%); carbohydrate transport and metabolism (7%); energy production and conversion (6%); lipid transport and metabolism (6%); replication, recombination and repair (5%); amino acid transport and metabolism (5%); defense mechanism (4%); chromatin structure and dynamics (4%); cytoskeleton (3%) and those with unknown function (5%).

Thirteen canonical pathways were obtained from IPA (Figure S2). Z-score value was used to predict the activation state of the upstream regulator. The absolute z-score above 2 is considered significant. According to z-score, orange bar indicates that the activity pattern of this pathway was predicted; blue bar indicates that the inhibition pattern of this pathway was predicted and grey bar indicates that the pathway can’t be assessed by this method. Ratio value indicates the number of molecules in a given pathway that meet cut criteria, divided by total number of molecules that make up that pathway^8^. Among the pathways, TCA Cycle II (eukaryotic) pathway (Figure S3) and NADH repair pathway (Figure S4) were associated with the energy metabolism of cardiomyocyte. Isocitrate dehydrogenase 3 (NAD(+)) alpha (IDH3A) was the significant and up-regulated protein in TCA Cycle II (eukaryotic) pathway. Glyceraldehyde-3-phosphate dehydrogenase (GAPDH) was the significant and up-regulated protein in NADH repair pathway.

**Figure 3:**
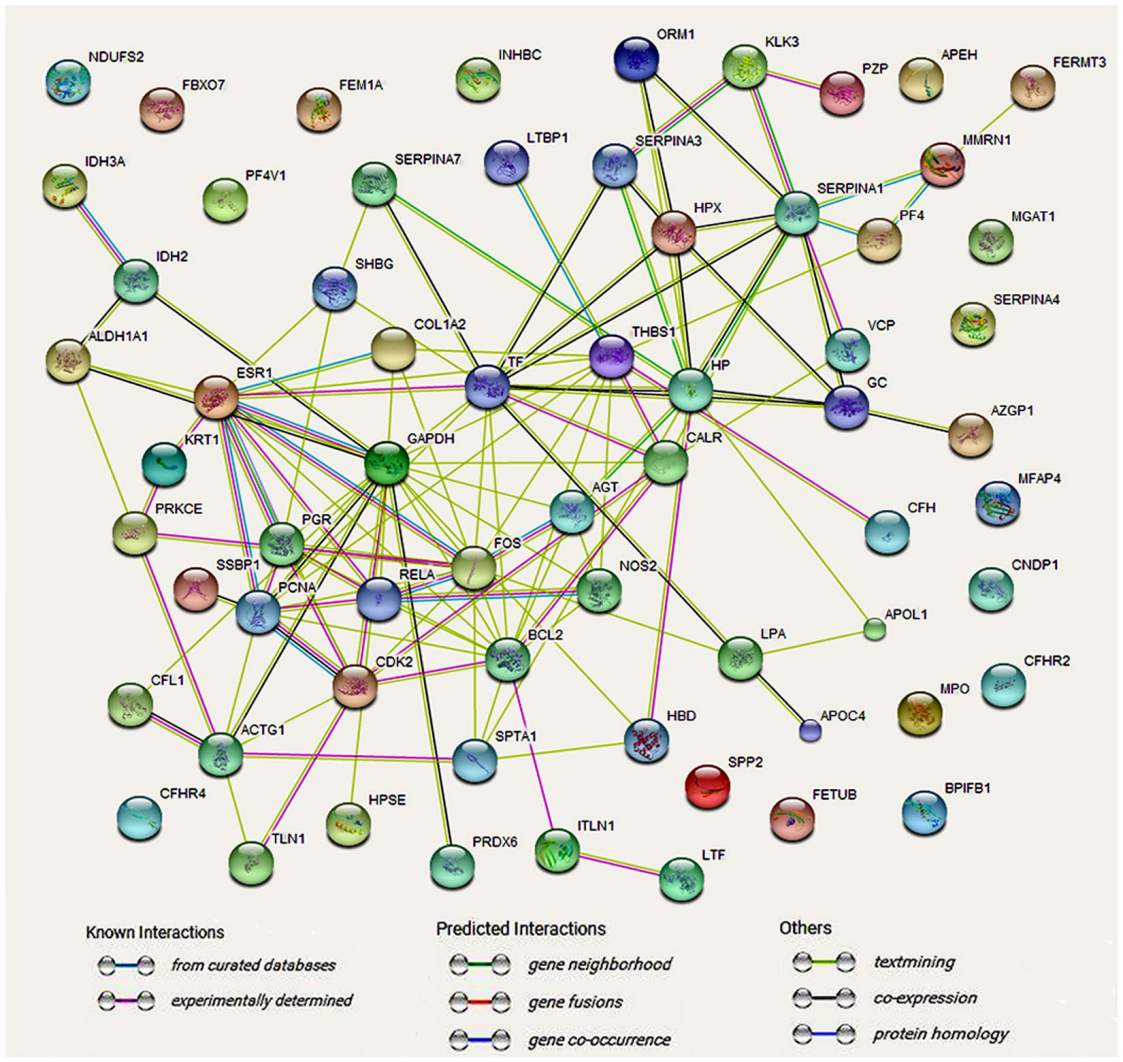
The interaction partners of the KD serous significant proteins network from STRING. Proteins are presented in the way of genes symbols. The implications of different colorful lines between the proteins are presented below the network map.

STRING successfully identified 65 differently expressed proteins and resulted in one network (Fig. 3). The most dense network of associations was observed around GAPDH, while calreticulin (CALR) had five, IDH2 three, SSBP1 one and IDH3A one experimentally determined relationships. Altogether 16 genes were single nodes, with no associated genes. IDH2 has relationship with IDH3A. GAPDH has relationship with CALR and IDH2.

## Discussion

KD is an endemic mitochondrial cardiomyopathy. The crista membranes of mitochondria in myocardium were swollen and enlarged. Meanwhile, the activity of oxidative phosphorylation enzymes was significant reduced in the mitochondria of KD patients^9^. Mitochondria-related gene expression profiles have been shown to be different between KD patients and healthy controls^10^.

These studies on interactions between multiple genes are becoming more and more meaningful than a single gene analysis in order to gain deeper understanding on the pathogenesis of complex diseases. Investigation of the genes that co-expressed in the same tissue with the target gene may be an important approach to identify the interacting genes. In this study, we used microarray to screen target genes associated with KD. Then, we combined the co-expression analysis and proteomics analysis to verify significant co-expressed genes and the proteins they encode with the target genes. Finally, we applied function categorization, pathway and network analysis to analyze these differently expressed proteins.

In our study, microarray analysis revealed that IDH2, FEM1A and SSBP1 were up-regulated genes in KD patient compared with the normal group. While proteomic iTRAQ analysis showed that proteins IDH2, FEM1A, SSBP1, CALR and NADH: ubiquinone oxidoreductase core subunit S2 (NDUFS2) were present at elevated level in KD patients’ serum. Genevestigator analysis predicted also CALR and NDUFS2 to co-express with IDH2 in peripheral blood mononuclear cell and cardiomyocyte, and their elevated protein levels in KD sera were confirmed by iTRAQ. Target gene analysis of FEM1A and SSBP1 gave no predicted co-expressed genes, which were common with the differences at protein level.

In mitochondria, IDH2 encodes isocitratedehydrogenase2, which catalyzes isocitric acid to generate α-ketoglutarate in tricarboxylic acid cycle and restores the NAD^+^ or NADP^+^ to NAD or NADP. These two processes are important for the energy metabolism, biological synthesis and anti-oxidative stress^11^. Simvastatin can decrease the NAD in myocardium of mice, which can relieve myocardial ischemia-reperfusion injury. In this study, IDH2 was up-regulation in KD than normal control. IDH2 caused the up-regulation of NAD, which may lead to the cardiomyopathy of KD.

FEM1A is also a mitochondrial protein, and preferentially expressed in organs enriched in mitochondria, such as cardiac muscle, brain and liver. It has also been observed that FEM1A expression is increased in hearts from mice subjected to ischemia–reperfusion injury. It is localized within mitochondria of C2C12 myoblasts and cardiomyocytes^12^. In this study, FEM1A was up regulated in KD, which indicates the mitochondrial dysfunction in KD may associate with FEM1A.

SSBP1 is believed to maintain the stability of genome, and coordinate the functions of DNA polymerase and the mitochondrial DNA helicase^13^. Over-expression of SSBP1 increased the amount of elongated or fragmented mitochondria in murine C2C12 myoblast cells. On the other hand, the silencing of SSBP1 by RNA interference led to an increase in fragmented or elongated mitochondria in the cell, suggesting that SSBP1 was involved in the processes of mitochondrial fusion and fission. The expression of SSBP1 changes may cause the abnormality of mitochondrial morphology and function^14^. Thus, it is obvious that the increased expression of SSBP1, as noticed in KD samples of this study, may cause the abnormalities of mitochondrial morphology and function.

CALR is a 46 kDa, ER-lumenal Ca2^+^-binding protein and molecular chaperone, which is required for proper heart development, and is induced in animal models of hypertrophic heart disease^15^. CALR expression increased in the pathologic heart, where it modulates hypertrophic growth, potentially reducing the impact of the pathology^16^. The pathological feature of KD is ventricular dilatation^17^, thus, the ventricular dilatation of KD may associate with elevated level of CALR noticed in this study.

NDUFS2 is located in the hydrophilic arm of mitochondrial respiratory chain complex I, close to the membrane domain^18^, and it has been shown that NDUFS2 mutation affects mitochondrial respiratory chain complex I enzymatic function^19^. In this study, NDUFS2 was up regulated in KD patients. The result indicates that the mitochondrial respiratory chain complex I enzymatic dysfunction in KD may be associated with NDUFS2.

In TCA Cycle II (Eukaryotic) pathway, IDH3A was up regulated. Among the mammalian, IDH3A is supposed to play a major role in mitochondrial isocitrate decarboxylation in the TCA cycle^20^. GAPDH is a multifunctional protein that also mediates cell death under oxidative stress. In NADP repair pathway, it was up regulated. It reported previously that the active-site cysteine (Cys-152) of GAPDH plays an essential role in oxidative stress-induced aggregation of GAPDH associated with cell death. In isolated mitochondria, aggregates of WT-GAPDH directly induced mitochondrial swelling and depolarization, whereas mixtures containing aggregates of Cys-152 GAPDH reduced mitochondrial dysfunction^21^. The dysfunction of mitochondria in KD patient is verified by many studies as mentioned above. The up regulated of IDH3Aand GAPDH may play roles in the dysfunction of mitochondria in KD.

IDH2 has relationship with IDH3A. IDH3A and IDH2 both belong to the isocitrate dehydrogenase gene family^22^. GAPDH has relationship with CALR and IDH2 separately in protein network. GAPDH and IDH2 are both associated with the function of mitochondria^23^. The interactions of GAPDH with IDH2 and CALR will be our next step study aims.

In conclusion, Genevestigator can forecast the protein expression of the co-expression genes CALR and NDUFS2 of IDH2 in the same tissue or organ. The genes IDH2, FEM1A, SSBP1, CALR, NDUFS2, IDH3A, GAPDH and their encoded proteins may play important roles in the pathogenesis of KD. In addition, TCA Cycle II (Eukaryotic) pathway and NADP repair pathway may also participate in the pathogenesis of KD. Overall, the gene microarray combined iTRAQ comparative proteomics analysis may be a beneficial approach to reveal the molecular mechanisms of other diseases.

## Materials and Methods

### Collection of peripheral blood samples

Patients with KD came from the KD-affected areas, which are Xunyi and Huangling Counties at Shaanxi Province of China. Clinical evaluation was according to the KD diagnosis criteria in China (WS/T 210–2011). Peripheral blood samples were collected from 112 KD patients (61 females and 51 males, 43–70 years old) and 112 normal controls (60 females and 52 males, 45–67 years old) were matched by age and gender. Each sample is 2.5 ml. Whole blood samples from 106 KD patients and 106 normal controls were collected in heparinized vacutainer tubes containing RNA stabilizing solution. Sera of other 6 KD and 6 normal controls were collected after centrifugation of whole blood, 3000 rpm, 5 min. All samples were stored at −80 °C.

The normal control subjects didn’t contain cardiovascular disease, hypertension or diabetes patients. Every subject involved in the investigation signed the informed consent. This investigation obtained the approval of Human Ethics Committee of Xi’an Jiaotong University, Xi’an, Shaanxi, China. We confirmed that all experiments were performed in accordance with the relevant guidelines and regulations.

### RNA extraction and microarray analysis

Peripheral blood mononuclear cell (PBMC) RNA was extracted from the peripheral blood of 106 KD and 106 normal controls. One hundred KD (53 females and 47 males, 43–70 years old) and 100 normal controls (52 females and 48 males, 46–66 years old) were used for the oligonucleotide microarray. The rest of the RNA samples were used for quantitative reverse transcription-polymerase chain reaction (qRT-PCR) analysis. The protocol of PBMC RNA extraction and oligonucleotide microarray analysis were done as previously described^24^. The oligonucleotide microarray contained altogether 78 probes for the 78 differentially expressed genes^5^. Fold changes ≥3 and ≤0.33 between KD and control samples were considered as up- and down-regulated genes, respectively.

### Quantitative RT- PCR

Six KD (4 females and 2 males, 45–60 years old) and 6 normal controls (4 females and 2 males, 45–58 years old) were matched by gender and age. RNA was extracted from the peripheral blood of all the samples. To validate the microarray results, three differently expressed genes isocitrate dehydrogenase [NADP(+)] 2, mitochondrial (IDH2), fem-1 homolog A (FEM1A) and single stranded DNA binding protein 1 (SSBP1) genes were selected as three differently expressed target genes for qRT-PCR. qRT-PCR was done by following the manufacturer’s recommended protocol (Invitrogen, Germany). The qRT-PCR data were log-transformed to ensure normal distribution and analyzed using paired *t*-tests.

### Co-expression gene forecast analysis

Genevestigator (https://genevestigator.com/gv/) is a powerful tools search engine for gene expression with advanced analysis possibilities, including the search for genes that are specifically expressed under certain conditions, and the search for groups of genes sharing similar expression patterns by means of clustering and biclustering algorithms^25^. In this study, the co-expression function of Genevestigator was used to find co-regulated genes with a gene of interest. IDH2, FEM1A and SSBP1 were chosen as target genes separately. Firstly, in order to find conditions relevant for the gene of interest, perturbations tool was applied. *P*-value 0.05 and fold-change 2 or 0.5 were selected as criteria. Secondly, in order to find other genes regulated under the same conditions, co-expression tool was used to find the top 30 genes most likely co-expressed with the target gene across these relevant conditions. Finally, hierarchical clustering tool was applied to cluster the top 30 co-expressed genes by similarity across anatomical parts.

### Mass spectrometry (LC-MS/MS) analysis

Sera of 6 KD (4 females and 2 males, 58–66 years old) and 6 normal controls (4 females and 2 males, 55–67 years old) were used for iTRAQ analysis. The total protein of each sample was extracted using ProteoMiner^TM^ Kits (Bio-Rad, USA) according to the instruction. One hundred μg total protein was taken from each sample. After digested with Trypsin Gold (Promega, USA), the tryptic peptides were processed using iTRAQ reagent (Applied Biosystems, USA) according to the manufacturer’s protocol. LC-20AB HPLC Pump system (Shimadzu, Japan) was used for SCX chromatography. TripleTOF 5600 System (AB SCIEX, USA) was used for data acquisition.

Mascot 2.3.02 (Matrix Science, UK) was used for protein identification and quantification. The protein abundance with fold changes ≤0.67 and ≥1.5 (KD patients/normal ones) was considered as down-and up- regulated ones, respectively.

### Function analysis

Functional annotations of the differently expressed proteins were conducted using Blast2 GO (Gene Ontology) program against the non-redundant protein database (NR, NCBI). GO is an international standardization system of gene function classification. It has three ontologies, which can describe molecular function, cellular component, and biological process. COG (Cluster of Orthologous Groups of proteins) database (http://www.ncbi.nlm.nih.gov/COG/) was used to classify and group these identified proteins.

IPA (Ingenuity Pathway Analysis, http://www.ingenuity.com) is a repository of biological interactions and functional annotations from many relations between proteins, genes, complexes, cells, tissues, metabolites, drugs and diseases. IPA selected sources and databases from NCBI databases (for instance, Entrez Gene, Ref-Seq, OMIM disease associations, miRNA–mRNA target databases, GWAS databases and KEGG)^24^. The function of “Canonical pathway” in IPA was used to analyze the significant pathway. The genes from the dataset that met the log ratio cut-off of 3were considered relevant to be included in the analyses. We chose pathways that were statistically significant with a *P*-value ≤ 0.05 using the Fisher’s exact test.

STRING database (functional protein association networks, http://string.embl.de/) provides a score for each gene-gene or protein-protein interaction, which is computed as the joint probability of the probabilities from the different evidence channels (e.g., protein interaction, fusion, co-expression, text mining), correcting for the probability of randomly observing an interaction^26^. All the differently expressed proteins were analyzed using STRING-10 server. An interactome network was built for these sets of proteins to find out protein-protein interaction and to predict functional associations.

### Data Availability

The datasets generated during the current study are available from the corresponding author on reasonable request.

## Electronic supplementary material

Figure S1,S2,S3,S4
