# Supplementary material for: Prediction of co-expression genes and integrative analysis of gene microarray and proteomics profile of Keshan disease
Source: Sci Rep. 2018 Jan 10;8:231. doi: 10.1038/s41598-017-18599-x (PMC5762769; doi:10.1038/s41598-017-18599-x)
Supplement: Supplementary file 1 — Figure S1,S2,S3,S4 [file 41598_2017_18599_MOESM1_ESM.doc]

# Prediction of co-expression genes and [integrative analysis of gene microarray and proteomics profile of](https://www.ncbi.nlm.nih.gov/pubmed/28056817)Keshan disease

Sen Wang^1#^, Rui Yan^2#^, Bin Wang^3^, Peiru Du^1^, Wuhong Tan^1*^, Mikko J. Lammi^1,4*^, Xiong Guo^1^

^1^School of Public Health, Health Science Center of Xi’an Jiaotong University; Key Laboratory of Trace Elements and Endemic Diseases, National Health and Family Planning Commission. Xi’an, Shaanxi, China.

^2^Department of Cardiology, the Second Affiliated Hospital, Health Science Center of Xi’an Jiaotong University. Xi’an, Shaanxi, China.

^3^Ordance Industrial Hygiene Research Institute, Xi’an, Shaanxi, China.

^4^Department of Integrative Medical Biology, University of Umeå, Umeå, Sweden.

^#^ These authors contributed equally to this work.

*Address correspondence to: Wuhong Tan and Mikko J. Lammi, No. 76 Yanta West Road, Xi’an, Shaanxi 710061, China. Tel: 86-029-82655229; Fax: 86-029-82655032. E-mail address: [tanwh@xjtu.edu.cn](mailto:tanwh@mail.xjtu.edu.cn), mikko.lammi@umu.se


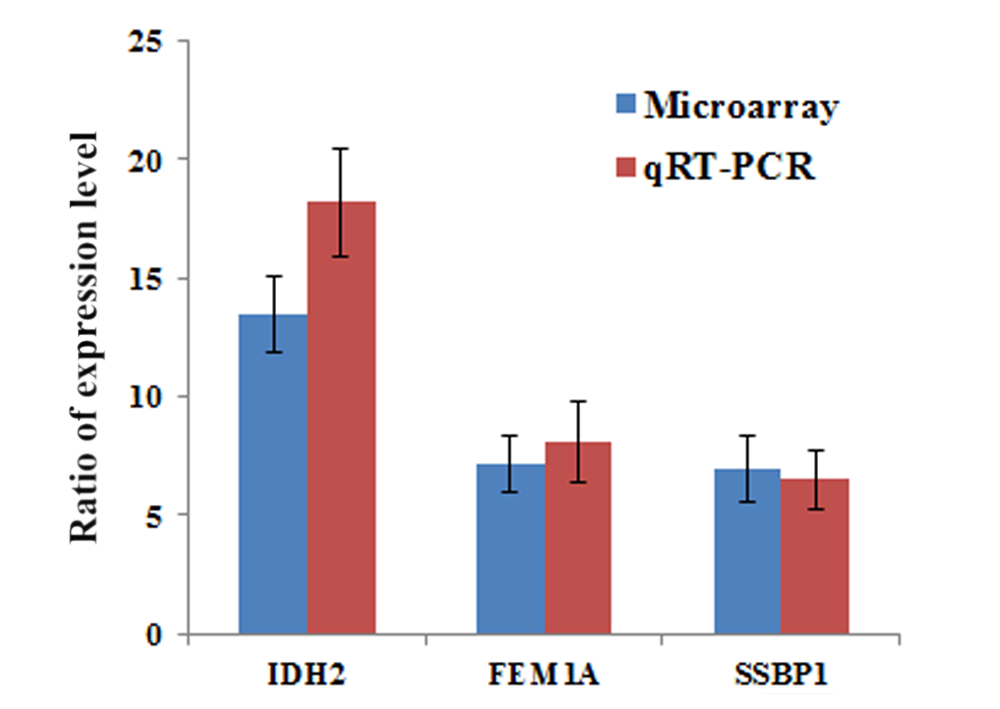


**Figure S1.** Bars indicate the expression values of selected genes measured by microarray (blue bars) and qRT-PCR (red bars) analyses. Values represent the mean± SD.


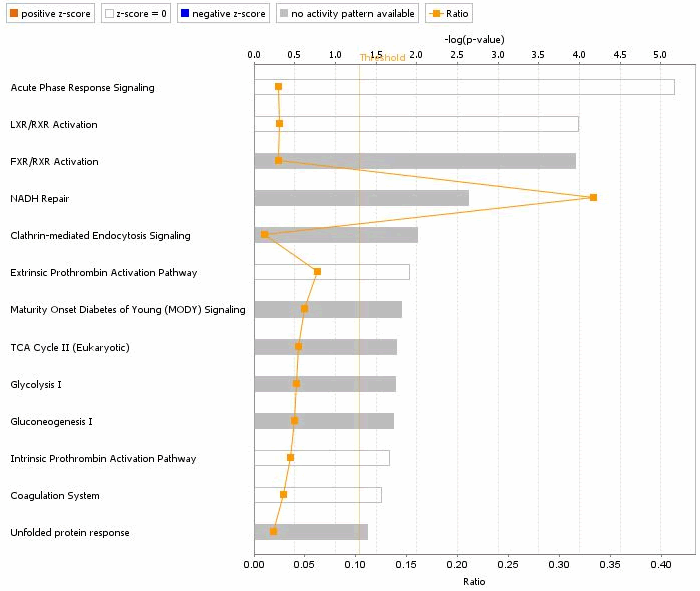


**Figure S2.** Canonical pathways of differently expressed proteins in serum of KD patients. Top functions that meet a *P*-value cutoff of 0.05 are displayed. The bar shows the –log (*P*-value) of each pathway.

**
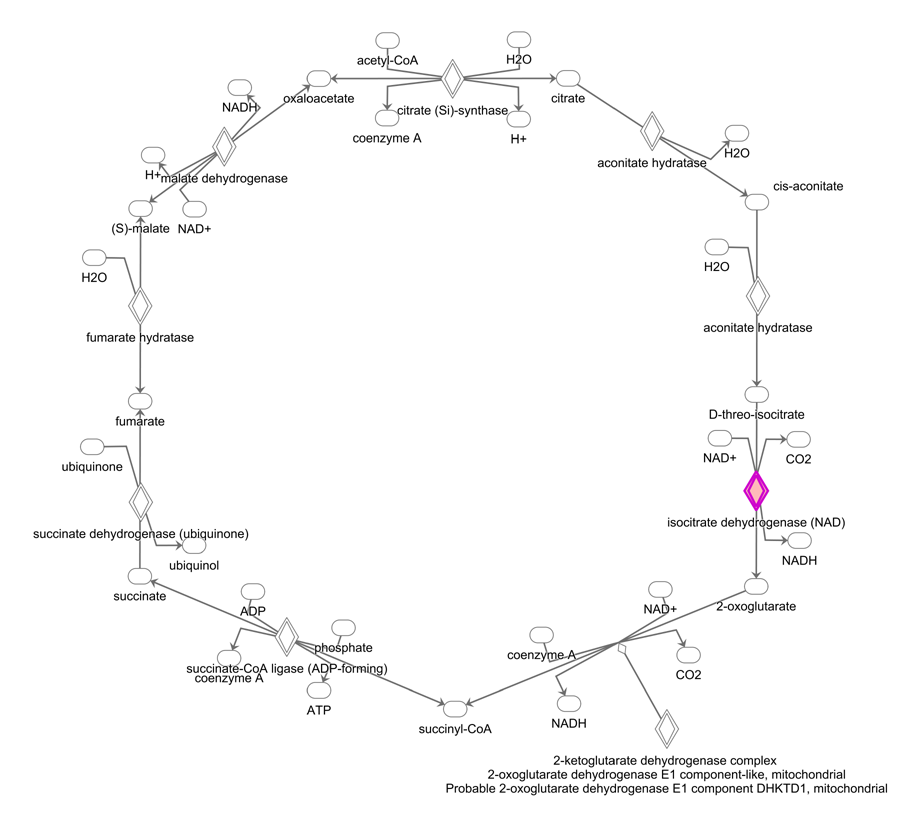
**

**Figure S3.** KD sera differently expressed proteins concerned in TCA Cycle II (Eukaryotic) pathway. Purple represents up-regulated protein and white symbols depict neighboring ones. Isocitrate dehydrogenase 3A (IDH3A) was up-regulated.

**
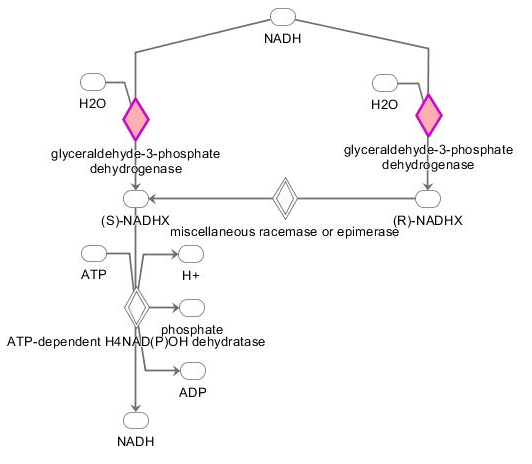
**

**Figure S4.** KD sera differently expressed proteins concerned in NADH repair pathway. Purple represents up-regulated protein and white symbols depict neighboring ones. Glyceraldehyde-3-phosphate dehydrogenase (GAPHD) was up-regulated.
